# Epac1 mediates thermogenesis and lipolysis in white adipose tissue via the p38γ-NFAT5 axis in a PKA-independent manner

**DOI:** 10.1042/CS20256710

**Published:** 2025-06-17

**Authors:** Baile Wang, Christina Yingxian Chen, Jie Liu, Qin Wang, Wenxia Zhang, Jingwen Liu, Andrew C.P. Tai, Alan Kai, Ben C.B. Ko, Aimin Xu, Sookja Kim Chung

**Affiliations:** 1 State Key Laboratory of Pharmaceutical Biotechnology, The University of Hong Kong, Hong Kong, China; 2 Department of Medicine, The University of Hong Kong, Hong Kong, China; 3 Guangdong-Hong Kong Joint Institute for Metabolic Medicine,The University of Hong Kong, Hong Kong, China; 4 School of Biomedical Sciences, The University of Hong Kong, Hong Kong, China; 5 Faculty of Medicine; Dr. Neher’s Biophysics Laboratory for Innovative Drug Discovery, State Key Laboratory of Quality Research in Chinese Medicine; Macau University of Science and Technology, Macao, China; 6 Department of Applied Biology & Chemical Technology, The Hong Kong Polytechnic University, Hong Kong, China; 7 Department of Pharmacology & Pharmacy, The University of Hong Kong, Hong Kong, China

**Keywords:** Epac1, lipolysis, NFAT5, p38γ, PKA-independent, thermogenesis, white adipose tissue

## Abstract

Beige adipocytes in white adipose tissue (WAT) share similar functions as brown adipocytes by converting lipids into heat through thermogenesis, while lipolysis is considered as a prerequisite for the activation of non-shivering thermogenesis. β3-adrenergic receptor (β3-AR) agonist CL316,243 (CL) and cold exposure are known to enhance lipolysis and beiging of WAT in a protein kinase A (PKA)-dependent manner, while the role of PKA-independent pathways involved is still poorly understood. Here, we show that the exchange protein directly activated by cAMP 1 (Epac1), a downstream target of cAMP, mediates β3-AR activation to modulate thermogenesis and lipolysis in a PKA-independent manner. Upon CL treatment or cold exposure, both thermogenic and lipolytic responses were compromised in Epac1-deficient mice, as evidenced by reduced oxygen consumption, less beige adipocytes, lower body temperature, and decreased circulating glycerol. Additionally, *in vitro* beige adipogenesis with or without cAMP analog treatment was significantly impaired in Epac1-deficient mice. Mechanistically, reduced total and phosphorylated p38γ and decreased induction of nuclear factor activated in T cells 5 (NFAT5) were observed in Epac1-deficient mice, which may contribute to the defective beiging of WAT. However, WAT of wildtype and Epac1-deficient mice showed no significant induction difference in phosphorylation of hormone-sensitive lipase at PKA and AMP-activated protein kinase sites with PKA activator, and *in vitro* beige adipogenesis was not altered in Epac1-deficient mice in response to PKA activation, indicating that Epac1 mediates lipolysis and beige adipogenesis in a PKA-independent manner. Taken together, Epac1 mediates β3-AR-induced beiging and lipolysis of WAT via the p38γ-NFAT5 axis in a PKA-independent manner.

## Introduction

Obesity, the result of energy intake exceeding energy expenditure, is an established risk factor for type 2 diabetes, cardiovascular disease, hypertension, and other related disorders [1]. When there is excessive energy intake, it is converted to lipid and stored in white adipose tissue (WAT). In contrast, brown adipose tissue (BAT) converts excessive lipids into heat and specializes in thermogenesis. There are two types of distinct thermogenic adipocytes that exist in rodents and humans: brown and beige (also called ‘brite’) [2]. While brown adipocytes are found predominately in dedicated BAT depots of rodents and infants, beige adipocytes sporadically distribute in WAT and develop in response to certain environmental cues, such as cold exposure and β3-adrenergic activation. This process is often referred to as ‘beiging’ of WAT. Uncoupling protein 1 (UCP1) is a signature protein expressed in these two types of thermogenic adipocytes. UCP1 is located on the inner mitochondrial membrane and dissipates excessive energy into heat by uncoupling oxidative phosphorylation from ATP synthesis, which results in non-shivering thermogenesis [3]. It has been found that the activity of BAT is inversely correlated to body mass index and age [4], indicating the vital role of thermogenesis in energy metabolism. Additionally, cold-induced BAT activation induces metabolic shifts (e.g., glucose lowering, white-to-brown fat transformation) to improve systemic metabolism by enhancing thermogenesis and suppress tumor growth by impairing glycolysis [5,6]. The BAT activation or expansion (via PDGFRα inhibition or miR-485 delivery) increases brown adipocyte numbers, counteracting obesity/diabetes and proposing BAT-targeted strategies (cold, drugs, or molecular interventions) as dual therapies for cancer and metabolic disorders [7]. Indeed, the enhancement of brown and beige fat has received considerable attention as a promising approach to counteract obesity and related disorders.

Studies have shown that treatment with the β3-adrenergic receptor (β3-AR) agonist CL 316,243 (CL) induces beige adipocytes and UCP1 expression in WAT in mice [8,9]. Protein kinase A (PKA) is activated as cAMP levels elevate upon β3-AR activation. The β3-AR induction of UCP1 in primary brown adipocytes requires activation of p38 mitogen-activated protein kinase (MAPK) in a PKA-dependent manner [10]. In addition to PKA, exchange proteins directly activated by cAMP (Epac1 and Epac2a, 2b and 2 c) have been identified as guanine nucleotide exchange factors for small GTPase Rap1 [11] and discovered to mediate PKA-independent cAMP signaling. For example, it has been reported that Epac plays a role downstream to the β-AR signaling in cardiomyocytes [12] and HEK-293 cells [13]. Nevertheless, it is not clear yet whether Epac mediates β3-AR signaling in adipose tissues.

In addition, β3-AR signaling is indispensable in the regulation of lipolysis [14], the process of triglyceride hydrolysis which releases glycerol and free fatty acids (FFA). Abnormal lipolysis is frequently associated with obesity and insulin resistance [15]. Hormone-sensitive lipase (HSL) is the rate-limiting enzyme in adipocyte lipolysis. The activity of HSL is regulated by several kinases. Phosphorylation of HSL by PKA [16] and extracellular signal-regulated kinase (ERK) [17] increases lipolysis, while phosphorylation by AMP-activated protein kinase (AMPK) shows the opposite effect [18]. It appears that the Epac-specific cAMP analog is able to induce part of the lipolysis in primary white adipocyte culture [19]. However, it has not been elucidated yet whether Epac1 or Epac2 mediates the increased cAMP by β3-AR activation in adipose tissues, since the analog activates both isoforms of Epac.

Previously, we demonstrated that Epac1-deficient mice exhibit a more severe high-fat diet-induced obesity and hyperglycemia phenotype than wildtype mice, suggesting the role of Epac1 in glucose, lipid homeostasis, and energy expenditure [20]. Even so, it has yet to be determined if such a metabolic phenotype is strictly due to Epac1 deficiency in β-cells, since Epac1 is also expressed by BAT and WAT, and Epac2 is not expressed by these tissues. We hypothesized that the metabolic phenotype of our Epac1-deficient mice may be the result of defects in fat metabolism as well. Therefore, we revisited the role of Epac1 in fat metabolism under normal physiological conditions, under the activation of β3-AR, and under cold exposure. In this study, we investigated the role of Epac1 in WAT beiging induced by β3-adrenergic activation and cold exposure by utilizing Epac1 knockout mice.

## Materials and methods

### Animal experiments

All experimental procedures related to live animals were approved by the Committee for the Use of Live Animals in Teaching and Research at The University of Hong Kong. Mice were kept on 12 hour light and dark cycles under controlled environmental settings (21 ± 1°C) with regular diet (RD, Lab Diet 5001) and water ad libitum. Age, sex, and genetic background-matched Epac1 knockout (Epac1^-/-^) mice [21], and wildtype (Epac1^+/+^) littermates were used in this study. Epac1^-/-^ mice were crossed with Epac2 knockout (Epac2^-/-^) mice [22] to generate Epac double-knockout (Epac1^-/-^; 2^-/-^) mice. Adipose tissues, liver, and brain were dissected from Epac1^-/-^; 2 ^/-^ mice for Western blot analysis of Epac1 and Epac2. To induce beiging of WAT, 8–12-week-old female or male Epac1^+/+^ and Epac1^-/-^ mice were treated with 10 consecutive intraperitoneal injections (i.p.) of CL (Tocris, Bristol, UK), at a dose of 1 mg/kg body weight [9], or subjected to short-term (6 hours) or long-term (48 hour) cold exposure at 6℃ after thermoneutralization at 30℃ for three weeks, respectively. Mice were killed by intraperitoneal injection of Pentobarbital (150–200 mg/kg body weight) before tissue harvest.

### Core temperature measurement

Core (rectal) temperature was measured by using a thermometer (YSI 4600, Dayton, OH) with a mouse rectal probe (YSI 451, Dayton, OH). Mice were anesthetized with a combination of ketamine (125 mg/kg body weight, i.p.) and xylazine (7.5 mg/kg body weight, i.p.) and kept on a heat pad set to 37°C to maintain body temperature. Rectal temperature was recorded every 2 minutes after a CL or saline injection for a period of 30 minutes. For mice subjected to 6hour- acute cold exposure at 6°C, core temperature was recorded every 2 hours from the start of cold exposure.

### Circulating FFA assay

Blood was collected by cardiac puncture and centrifuged at 3300 rpm to separate serum for the FFA assay. Blood fatty acid was measured by using a TRINDER modified enzyme-coupled colorimetric end-point assay automated on the Hitachi-912 analyzer (Roche Diagnostics Co., Basel, Switzerland).

### Metabolic studies

Indirect calorimetry was performed on acclimated, singly housed, 11–12 weeks old female Epac1^+/+^ and Epac1^-/-^ mice using a computer-controlled, open-circuit system (Oxymax System, Columbus Instruments, Columbus, OH), which is a part of an integrated Comprehensive Lab Animal Monitoring System (CLAMS; Columbus Instruments, Columbus, OH). Sample air was sequentially passed through O_2_ sensors (Columbus Instruments) for the determination of O_2_ content, from which measures of oxygen consumption (VO_2_) were estimated. Mice were allowed an overnight adaptation to the novel environment, and data acquired from the second day to the end of the experiment were analyzed. Epac1^+/+^ and Epac1^-/-^ mice were monitored in CLAMS for two to three days after adaptation. Saline-injected mice were tested to rule out any effect of handling and injection on VO_2_. Since there was no significant increase in VO_2_ induced by injection, the VO_2_ under normal conditions served as the control for acute CL injections. VO_2_ change induced by cold exposure for Epac1^+/+^ and Epac1^-/-^ mice was also recorded on a 12-hour day and night basis.

### Histology and immunocytochemistry

Peri-uterine WATs and interscapular BATs were dissected and fixed in neutral-buffered 10% formalin for around 48 hours, followed by 3 washes in phosphate-buffered saline (PBS) for 30 minutes each. The fixed tissues were dehydrated in an ascending series of ethanol (70%, 90%, 95%, and 100% ×3) and chloroform prior to embedding in paraffin for histological assessment [23]. For hematoxylin–eosin (H&E) and immunostaining, each paraffin-embedded tissue was cut into 4-μm-thick sections by using a microtome. The sections were mounted on microscopic slides coated with 3-aminopropyltriethoxysilane (Sigma, St. Louis, MO). To determine adipocyte area, three fields of view under the 20× objective on each H&E-stained section were analyzed. Immunostaining with antibodies against UCP1 (1:1000, Abcam #ab10983, Cambridge, MA) and nuclear factor of activated T-cells 5 (NFAT5) (1:1000, a kind gift from Prof. H. M. Kwon, University of Maryland) was performed on 4 μm paraffin sections using indirect avidin–biotin–peroxidase complex method (ABC kit, Vector Laboratories, Burlingame, CA). Immunoreactive signals were developed in 3,3-diaminobenzidine (DAB) peroxidase substrate solution for 2 minutes. The sections stained with the anti-UCP1 antibody were counter-stained with hematoxylin before mounting. Images were captured with a Spot RT Color CCD digital camera connected to an inverted microscope.

### Lipolysis assay in WAT explants *ex vivo*

Peri-uterine WAT was dissected from 10-week-old female Epac1^+/+^ and Epac1^-/-^ mice under fed conditions. WAT was cut into small portions (10 mg each) and rinsed in warm PBS twice. Each portion of WAT was further cut into 8–10 small cubes (1–2 mg each). These explants were transferred into 100 μL incubation buffer (Phenol red-free DMEM with 5 mM glucose and 0.5% FFA-free BSA) in a 96-well plate, washed for 30 min on a rocker, and then transferred into fresh wells with 50 μL incubation buffer. CL treatment solution (50 μl 2× of final concentration, 2 μM and 200 μM) or control (50 μl saline) was added to each well. Immediately, the incubation medium (6.5 μl from each sample) was collected for baseline readings (0 min). The plate was then transferred into the incubator with 5% CO_2_ at 37 ℃. The incubation medium was sampled at 30, 60, 120, and 240 min. Glycerol release was determined by using a glycerol assay (free glycerol reagent, Sigma, St. Louis, MO). WAT explants treated with CL (10 μM) or saline for 10 min and cold exposure for 2 hours were collected for Western blot analysis.

### Western blot analysis

Protein was extracted from adipose tissues by using a lysis buffer containing 50 mM Tris-HCl (pH 7.4), 150 mM NaCl, 1% NP-40, 0.25% Sodium deoxycholate, 0.1% SDS, 1 mM EDTA, 1 mM DTT, 1 mM PMSF with protease inhibitor cocktail (Roche Applied Science, Mannheim, Germany). Protein concentration was determined using a protein assay dye reagent concentrate (Bio-Rad, Hercules, CA). Protein samples (20 μg/well) were loaded and separated by 8–10% sodium dodecyl sulfate–polyacrylamide gel electrophoresis. The primary antibodies used for Western blot analyses included rabbit anti-UCP1 (1:1000, Abcam, Cambridge, MA), phosphor-AMPK Thr172 (kindly provided by Dr. Y.P. Ching, Hong Kong), PKA regulatory subunit 1 alpha (1:1000, Abcam, Cambridge, MA), phospho-PKA-RII (Ser96) (1:1000, ABT58, EMD Millipore, Darmstadt, Germany), phospho-p38 MAPK, total p38α, total p38γ, Phospho-p44/42 MAPK (pERK1/2) and p44/42 MAPK (ERK1/2) (1:1000, Cell Signaling Technology, Danvers, MA), Phospho-HSL (Ser660) and HSL, sheep anti-AMPK (kindly provided by Dr. Y.P. Ching, Hong Kong), and mouse anti-α-tubulin (1:4000, Sigma, St. Louis, MO), anti-β-tubulin (1:2500, Cell Signaling Technology, Danvers, MA), Epac1 and Epac2 (kindly provided by Professor J. Bos from the UMC Utrecht) antibodies. Bound antibodies were visualized with horseradish peroxidase-conjugated secondary antibodies, and immunoreactivity was assessed by chemiluminescence reaction, using the ECL Western detection system (GE Healthcare). Densitometric analysis was performed to quantify protein expression (ImageJ, U. S. National Institutes of Health, Bethesda, MD).

### qPCR analysis

Total RNA was isolated from snap-frozen adipose tissues and adipocyte cultures using TRI REAGENT (Molecular Research Center, Inc., Cincinnati, OH) following the manufacturer’s instructions. Total RNA (1 μg) was treated with RNase-Free DNase and reverse transcribed using a transcriptor first strand cDNA synthesis kit (Transcriptor reverse transcriptase, Roche Applied Science, Mannheim, Germany). Each cDNA sample was analyzed by real-time qPCR assays on the iQ^TM^ 3 detection system (Bio-Rad, Hercules, CA) using the SYBR Green Supermix (Bio-Rad). The expression levels of each target gene were normalized to the TATA-box binding protein (TBP) mRNA and expressed in arbitrary units (see Table 1 for primer sequences).

**Table 1:** Sequences of primers used in qPCR analysis.

	Primer sequences	
Gene	Forward	Reverse
UCP1	5’-GGCATTCAGAGGCAAATCAGCT-3’	5’-CAATGAACACTGCCACACCTC-3’
Cidea	5’-GTCGCCAAGGTCGGGTCAAGTC-3’	5’-AAAGGGCGAGCTGGATGTATGAGG-3’
PGC1α	5’-GATGCGCTCTCGTTCAAGAT-3’	5’-GGTGTCTGTAGTGGCTTGAT-3’
PPARγ	5’-AGGCGAGGGCGATCTTGACAG-3’	5’-AGGGCTTCCGCAGGTTTTTGA-3’
CEBPα	5’-CAAGAACAGCAACGAGTACCG-3’	5’-GTCACTGGTCAACTCCAGCAC-3’
TBP	5’-TGCACAGGAGCCAAGAGTGAA-3’	5’-CACATCACAGCTCCCCACCA-3’

### Isolation and adipose differentiation of mouse embryonic fibroblast (MEF)

Epac1^+/+^ and Epac1^-/-^ embryos were dissected from the uterus on embryonic day 14.5. The extra-embryonic membranes and viscera were removed. Then, the rest of the embryo was cut into small pieces and incubated in 4 ml of 0.25% trypsin-EDTA for 30 min at room temperature. Trypsin was neutralized by adding Dulbecco’s modified Eagle’s medium (DMEM) high glucose with 10% fetal bovine serum (FBS), 100 U of penicillin, and 100 µg of streptomycin per ml. The cell suspension was passed through a 40 μm cell strainer to remove undigested tissues. Then, the cell suspension was pelleted at 1200 rpm for 4 minutes. The supernatant was aspirated, and fresh medium was added to suspend the cell pellet. The MEFs were plated at a density of 1.5 × 10^4^ /cm^2^. Two days after confluence (day 0), cells were treated with DMEM high glucose containing 10% FBS, 0.5 mM IBMX, 1 μM dexamethasone, 5 μg/ml insulin, and 25 μM pioglitazone for two days. On differentiation day 3, cells were switched to the maintenance medium, DMEM high glucose containing 5 μg/ml insulin and 25 μM pioglitazone. The maintenance medium was refreshed every two days. Cells were harvested for oil red O staining (Sigma, St. Louis, MO) or total RNA isolation for qPCR analysis, at different time points as indicated in the results.

### Isolation and differentiation of stromal vascular fractions

Subcutaneous WATs from eight-week-old male Epac1^+/+^ and Epac1^-/-^ mice or BATs from five-week-old Epac1^+/+^ and Epac1^-/-^ mice were minced into pieces with sterile scissors before digesting in DMEM containing 2 mg/ml of Collagenase type I (Gibco, #17100017) and 3% of bovine serum albumin (BSA, Sigma, #A7906) for 30 min at 37°C. The digested tissues were then filtered through a 70 μm cell strainer (BD Biosciences) and centrifuged at 800 × g for 10 min at 4°C to separate SVFs from the mature adipocyte fraction. The isolated SVFs were then resuspended with DMEM and plated on a 10 cm cell culture dish. After reaching 90% confluency, cells were subcultured and seeded in a 24-well plate (5 × 10^4^ cells per well) until they reached 100% confluency. For white adipocyte differentiation, induction medium containing 1 μM dexamethasone, 0.5 mM isobutylmethylxanthine (IBMX), 1 μM rosiglitazone, and 3 IU/mL insulin was added to each well. After 48 hours, the medium was changed to a maintenance medium containing 1 μM rosiglitazone and 1.8 μM insulin. The maintenance medium was replaced every 2 days until day 8. For beige adipocyte differentiation, induction medium containing 1 µM dexamethasone, 0.5 mM IBMX, 1 µM rosiglitazone, 0.125 µM indomethacin, 1 nM T3, and 3 IU/mL insulin was added to each well. After 48 hours of induction, the medium was changed to a maintenance medium containing 1 µM rosiglitazone, 1 nM T3, and 3 IU/mL insulin. The maintenance medium was replaced every two days until day 8. Cells were harvested after 8 day differentiation and then subjected to RNA extraction for further qPCR analysis. To evaluate the role of cAMP and PKA signaling pathways on beige differentiation, 3 µM forskolin (MCE, #HY-15371) or 1 mM cAMP analog (8-bromo-Cyclic AMP, Cayman, #CAY14431) was added into beige induction medium to treat the cells for 48 hours.

### Statistical analysis

Unless stated, data are presented as mean ± SEM. Unpaired Student’s *t*-test or two-way ANOVA followed by post-hoc Tukey’s multiple comparisons test was performed to determine statistical significance (GraphPad Prism software, San Diego, CA). A test with *P* < 0.05 was considered statistically significant.

## Results

### Epac1 is the dominant form of Epac proteins expressed in WAT, BAT, and liver

**Figure 1:**
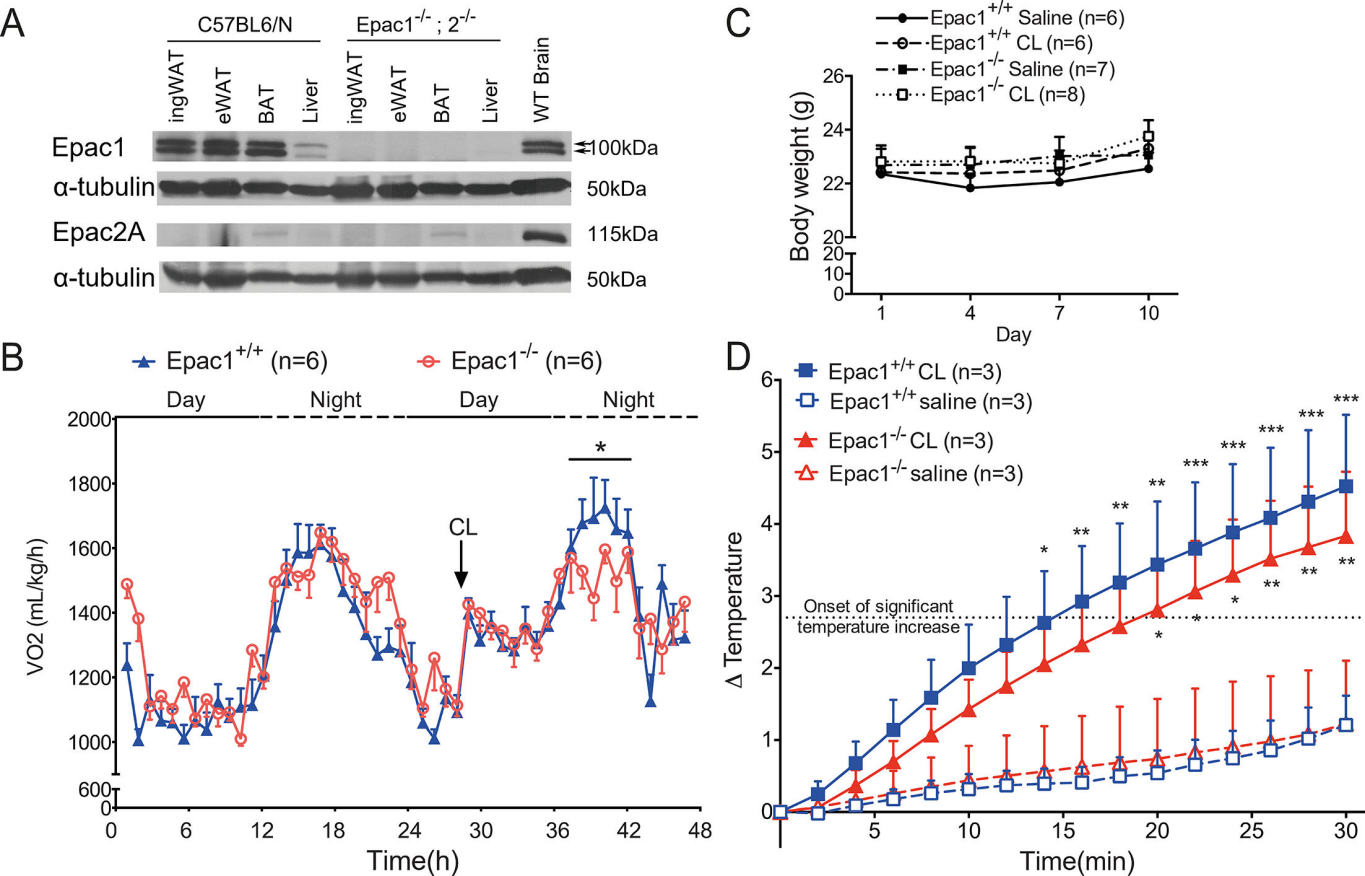
**Epac1 mediates β3-AR agonist (CL)-induced oxygen consumption and thermogenesis**. (**A**) Western blot of Epac1 and Epac2 in inguinal WAT (ingWAT), epididymal WAT (eWAT), and interscapular BAT (BAT). Expression of Epac1 and Epac2 in brain as positive controls. (**B**) Oxygen consumption (VO_2_) before and after CL treatment measured by CLAMS (*n* = 6). (**C**) Body weight of Epac1^+/+^ and Epac1^-/-^ mice during CL or saline treatment (*n* = 6–8). (**D**) Increase in core temperature of mice after saline or CL treatment (*n* = 3). The dotted line represents the onset of significant temperature increase relative to the saline treated conditions. Data are presented as mean ± SEM. *P<0.05, **P<0.01, ***P<0.005 between saline and CL-treated mice in each genotype. β3-AR, β3-adrenergic receptor; BAT, brown adipose tissue; WAT, white adipose tissue.

Western blot analysis showed that Epac1 was abundantly expressed in epididymal and inguinal WATs, BAT, and liver (Figure 1A), while Epac2 was not detected in these tissues (Figure 1A). Protein extracts from the brain were used as positive controls for both Epac1 and Epac2 expression [20,24]. The faint band in BAT is likely due to antibodies cross-reacting with another protein, as it is also present in the Epac1^-/-^;2 ^/-^ mice. Therefore, the role of Epac1 in β3-adrenergic activation in adipose tissues was further investigated by comparing the wildtype and Epac1^-/-^ mice.

### CL treatment-induced oxygen consumption and thermogenesis are compromised in Epac1^-/-^ mice

Given the vital role of β3-AR signaling in lipid metabolism and energy expenditure, oxygen consumption (VO_2_) induced by acute CL treatment (1 mg/kg b.w., i.p.) was assessed by indirect calorimetry. After CL treatment, Epac1^+/+^ mice showed a significant increase in VO_2_ at nighttime compared with that of the controls, whereas the VO_2_ increase in the Epac1^-/-^ mice was lower (Figure 1B, *P* = 0.0378 by two-way ANOVA), indicating that Epac1 plays a role in β3-AR activation induced energy expenditure.

Additionally, we also examined core temperature in mice those received multiple injections of CL. Over the course of 10 day injections, the body weight of Epac1^+/+^ and Epac1^-/-^ mice was similar (Figure 1C). Saline-treated Epac1^+/+^ and Epac1^-/-^ mice showed similar changes in core temperature (Figure 1D). CL induced a smaller increase in core temperature in Epac1^-/-^ mice compared to Epac1^+/+^ mice but not to a significant level. However, Epac1^-/-^ mice only show a significant temperature rise 20 minutes after CL treatment, which is relatively delayed compared to Epac1^+/+^ mice (14 min after CL treatment), demonstrating that Epac1 is involved in thermogenesis induced by β3-AR activation.

### Epac1^-/-^ mice show defects in β3-AR-activated beiging of WAT

**Figure 2:**
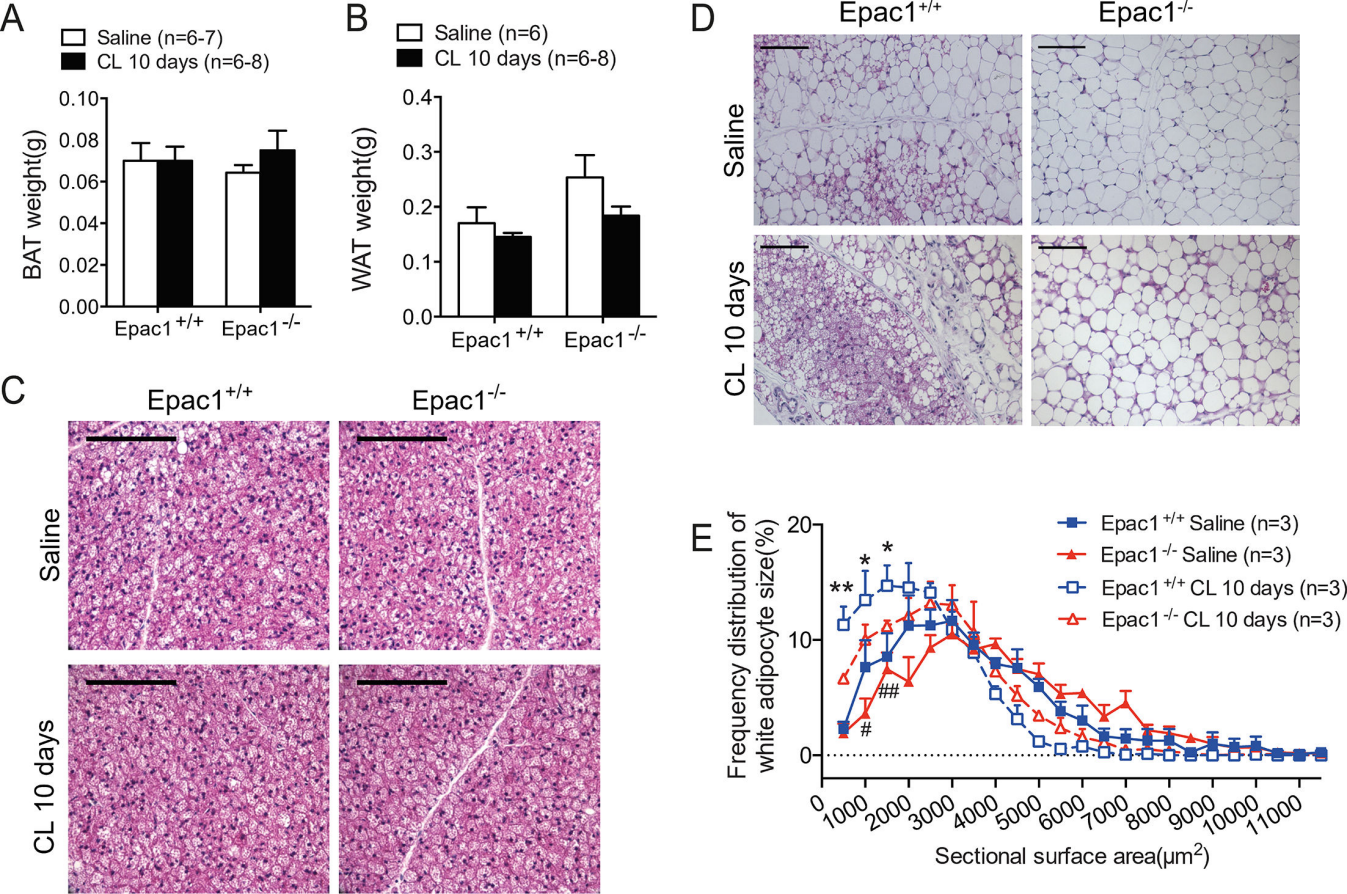
**Epac1^-/-^ mice show defects in β3-AR-stimulated beiging of WAT**. (**A**) BAT weight and (**B**) peri-uterine WAT weight after CL or saline treatment for 10 days (*n* = 6–8). (**C-D**) Representative images of H&E stained BAT (**C**) and WAT (**D**) from Epac1^+/+^ and Epac1^-/-^ mice with CL or saline for 10 days. Bar = 100 μm (*n* = 4–5). (**E**) Distribution curves of adipocytes area in WAT. Statistical significance between Epac1^+/+^ and Epac1^-/-^ in CL (*) or saline (#) treatment is shown. Data are presented as mean ± SEM. CL vs. saline in Epac1^+/+^ mice group, *P<0.05, **P<0.01; CL vs. saline in Epac1^-/-^ mice group, #*P*<0.05, ##*P*<0.01. BAT, brown adipose tissue; WAT, white adipose tissue.

**Figure 3:**
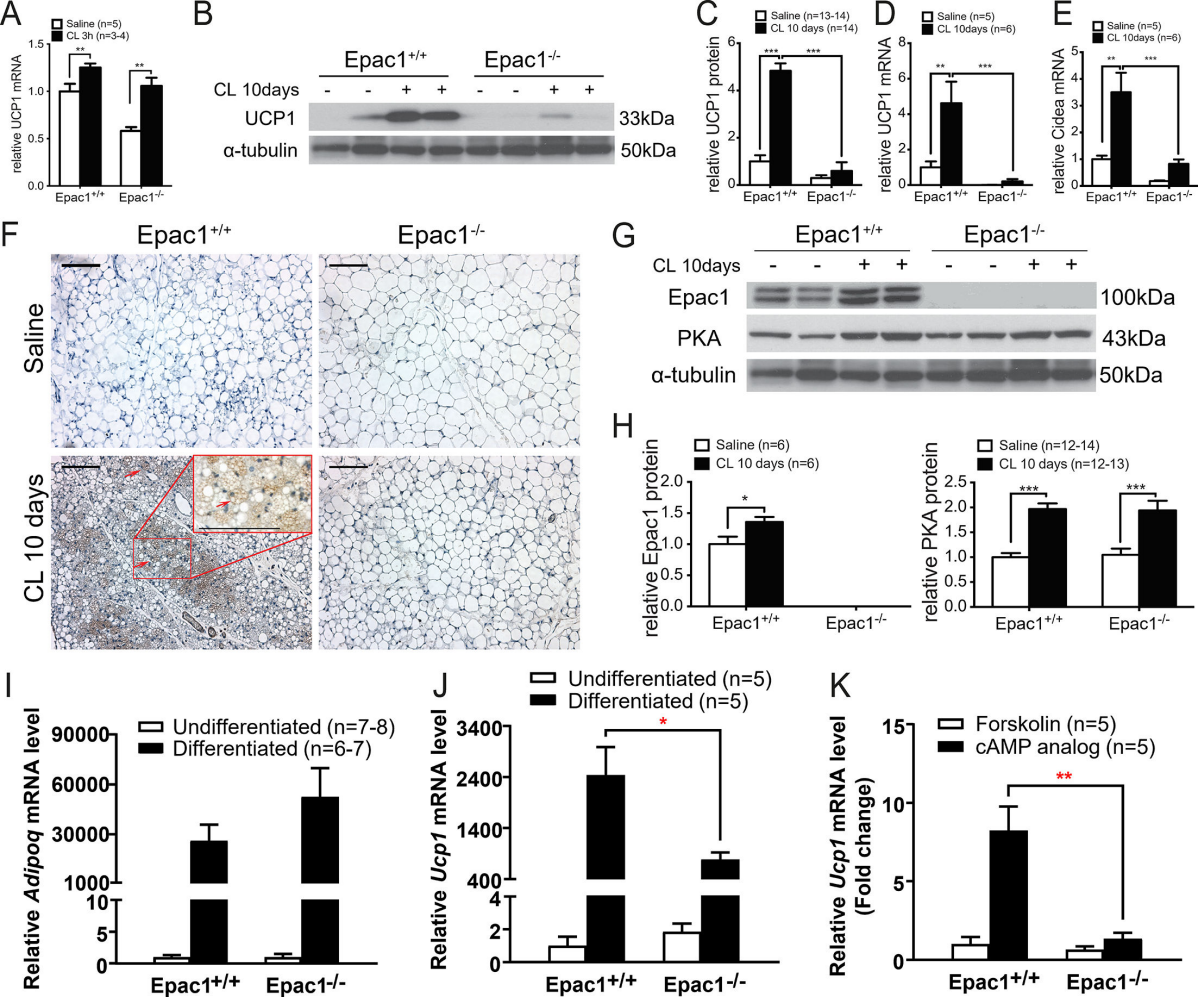
**The impaired CL-induced UCP1 expression and beige adipocyte differentiation in Epac1^-/-^ mice are independent of PKA**. (**A**) UCP1 mRNA levels in the BAT of Epac1^+/+^ and Epac1^-/-^ mice 3 hours after CL injection (*n* = 3–5). (**B**) Western blot analysis of UCP1 protein expression in WAT after CL treatment for 10 days (*n* = 13–14). (**C**) Quantification of UCP1 protein in WAT using densitometric analyses. (**D**) UCP1 and Cidea mRNA expression in WAT after CL treatment for 10 days (*n* = 5–6). (**F**) UCP1 immunostaining in WAT (*n* = 4–5). Arrows indicate beige cells with multilocular lipid droplets and rounder nuclei in Epac1^+/+^ CL 10 days, as shown in the high-magnification inset. Bar = 100 μm. (**G-H**) Western blot analysis of Epac1 (G, *n* = 6) and PKA (H, *n* = 12–14) protein expression in WAT by CL for 10 days (**G**) and quantification of Epac1 and PKA protein in WAT using densitometric analyses (**H**). (**I-K)** Stromal vascular fractions (SVFs) were isolated from ingWAT of 6-week-old Epac1^+/+^ and Epac1^-/-^ mice and were then differentiated into white or beige adipocytes. (**I**) mRNA level of Adipoq in differentiated white adipocytes derived from SVF. *n* = 6–8. (**J**) mRNA level of UCP1 in differentiated beige adipocytes derived from SVF. *n* = 5. (**K**) SVFs were treated with 3 μM forskolin or 1 mM cAMP analog (8-bromo-Cyclic AMP) in a beige induction medium. qPCR analysis of UCP1 mRNA levels were presented as fold change. Data are presented as mean ± SEM. *P<0.05, **P<0.01, ***P<0.001. PKA, protein kinase A; SVFs, stromal vascular fractions; WAT, white adipose tissue.

Due to the primary role of BAT in thermogenesis, we asked whether the abovementioned reduced oxygen consumption and thermogenesis originate from defects in BAT. However, there was no difference in the weights (Figure 2A) and morphology (Figure 2C) of BAT between Epac1^+/+^ and Epac1^-/-^ mice treated with saline and CL for 10 days. Similar to Epac1^+/+^ control mice, CL could also significantly induce UCP1 mRNA level in the BAT of Epac1^-/-^ mice (Figure 3A), suggesting that the compromised CL-induced oxygen consumption in Epac1^-/-^ mice was not likely caused by a defect in BAT. Given that CL-induced UCP1 mRNA level in BAT was similar in Epac1^+/+^ and Epac1^-/-^ mice, the compromised CL-induced VO_2_ in Epac1^-/-^ mice could be attributed to defects in downstream signals of β3-AR in WAT. Saline-treated Epac1^-/-^ mice had a slightly larger mass of peri-uterine WAT than that of Epac1^+/+^ mice, although the difference was not significant (Figure 2B). The weight of peri-uterine WAT in both Epac1^+/+^ and Epac1^-/-^ mice showed a trend of decrease after 10 days of CL treatment (Figure 2B), but it was not significant. The peri-uterine WAT of CL-treated mice demonstrated a beige fat phenotype, that is, smaller eosinophilic adipocytes containing multilocular lipid droplets (Figure 2D). Interestingly, WAT in CL-treated Epac1^-/-^ mice showed less beige fat compared with that of CL-treated Epac1^+/+^ mice (Figure 2D). Cell size distribution curve (Figure 2E) shows that the Epac1^-/-^ WAT contained a lower number of small adipocytes (sectional area of 1000–2000 μm^2^) compared with that of Epac1^+/+^ under basal conditions. CL treatment for 10 days reduces adipocyte size, as indicated by the left shift of the distribution curve of adipocyte size in both genotypes (Figure 2E). The fraction of small adipocytes (sectional area <2000 μm^2^) in CL-treated Epac1^-/-^ WAT was significantly lower than that of Epac1^+/+^ WAT (Figure 2E), suggesting that the absence of Epac1 reduces the effect of CL on WAT remodeling.

### Ablation of Epac1 impairs CL-induced UCP1 expression

The reduced beiging of WAT in Epac1^-/-^ mice is further confirmed by Western blot analysis (Figure 3B and C) and immunostaining of UCP1 (Figure 3F). After CL treatment for 10 days, UCP1 expression was up-regulated (Figure 3B, C and F) concomitantly with CL-induced beige fat phenotype in the Epac1^+/+^ WAT (Figure 2D), both of which were suppressed in the absence of Epac1. In addition, mRNA levels of UCP1 (Figure 3D) and another BAT-enriched gene Cidea (Figure 3E) were less induced by CL treatment in Epac1^-/-^ WAT. In wildtype mice, both Epac1 and PKA (Figure 3G and H) expression were increased in WAT after 10 days of CL treatment. Interestingly, PKA expression in Epac1^-/-^ WAT was increased by CL to a similar level of Epac1^+/+^, yet there was less beige fat induced in WAT, suggesting the different ability in beiging is not likely due to differential PKA expression.

### Epac1 mediates *in vitro* differentiation of beige adipocytes in a PKA-independent manner

To further investigate whether Epac1 mediates the *in vitro* differentiation of beige or white adipocytes, we isolated stromal vascular fractions (SVFs) from ingWAT of Epac1^+/+^ and Epac1^-/-^ mice to differentiate them into white and beige adipocytes, respectively. We found that depletion of Epac1 has no impact on white adipocyte differentiation but significantly impaired the differentiation of beige adipocytes (Figure 3I and J). Next, we explored the effect of cAMP analog (8-bromo-Cyclic AMP, an activator of Epac1) and PKA activator (forskolin) on beige adipocyte differentiation upon Epac1 deficiency. Real-time PCR analysis of UCP1 showed that the cAMP analog could significantly induce beige adipocyte differentiation in SVFs from Epac1^+/+^ mice, while this induction was abolished in SVFs from Epac1^-/-^ mice (Figure 3K). Upon the treatment of the PKA activator forskolin, no obvious change was observed in SVF-derived beige adipogenesis of Epac1^-/-^ mice compared to Epac1^+/+^ mice (Figure 3K), indicating that Epac1 mediates beige differentiation in a PKA-independent manner.

### Impaired β3-AR stimulation of lipolysis in Epac1^-/-^ WAT is independent of PKA/HSL, ERK1/2, and AMPK signaling

**Figure 4:**
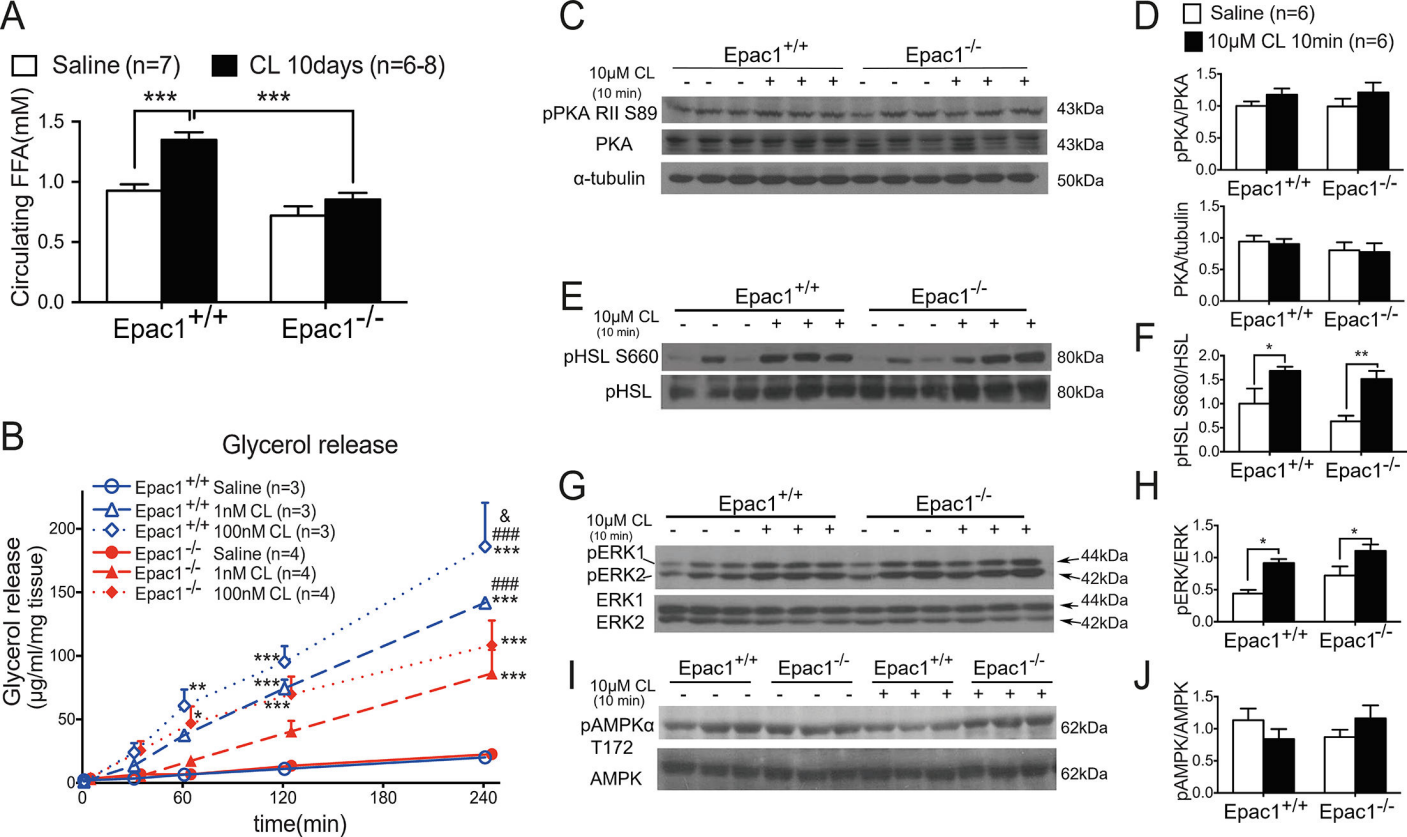
**Impaired CL-induced lipolysis in Epac1^-/-^ WAT is independent of HSL phosphorylation mediated by PKA**. (**A**) Circulating FFA after 10 days of CL treatment (*n* = 6–8). (**B**) Glycerol release from WAT explants incubated with saline, 1 nM or 100 nM CL. CL vs. saline: **P<0.01, ***P<0.001. 100 nM CL vs. 1 nM CL: &*P*<0.01. Epac1^+/+^ vs. Epac1^-/-^: ###*P*<0.001. *n* = 3–4. (**C-J**) Western blot analysis of PKA, pPKA RII S89 (**C**), phospho-HSL at Ser660 (**E**), pERK1/2 (**G**) and pAMPKα (T172) (**I**) in WAT explants (*n* = 6). Ratios of phospho-protein to total protein levels are quantified (**D, F, H, J**). Data are presented as mean ± SEM. *P<0.05, **P<0.01, ***P<0.001. FFA, free fatty acid; HSL, hormone-sensitive lipase; PKA, protein kinase A; WAT, white adipose tissue.

CL treatment-induced lipolytic activity in mice, manifested in increased circulating FFA levels [25,26], is reported to play a crucial role in the metabolic plasticity of WAT [27], by increasing mitochondrial biogenesis and oxidative capacity. We investigated whether this remodeling mechanism is attenuated in the absence of Epac1. Saline-treated Epac1^+/+^ and Epac1^-/-^ mice exhibited similar FFA levels in the serum (Figure 4A). Interestingly, CL treatment for 10 days led to a significantly higher increase in FFA levels of Epac1^+/+^ mice, while this obvious increase was absent in Epac1^-/-^ mice (Figure 4A). To examine whether WAT is contributing to CL-induced circulating FFA levels, we measured glycerol release from Epac1^+/+^ and Epac1^-/-^ WAT explants incubated in saline, 1 nM or 100 nM CL at different time points. CL-induced glycerol release from WAT explants was increased in a dose and time-dependent manner in both genotypes (Figure 4B). Consistent with lower CL-induced FFA levels *in vivo*, Epac1^-/-^ WAT explants exhibited less glycerol release relative to that of the Epac1^+/+^, indicating the role of Epac1 in the β3-AR stimulated lipolysis in WAT.

Given that lipolysis is increased when HSL is activated via phosphorylation of residue Ser660 mediated by PKA [16], we next tested if PKA/HSL activation was altered due to the deletion of Epac1. Between Epac1^+/+^ and Epac1^-/-^ WAT explants, there was no difference in the levels of PKA expression and phosphorylation of PKA (Figure 4C and D). In addition, HSL Ser660 phosphorylation was increased to similar levels in both Epac1^+/+^ and Epac1^-/-^ WAT explants after 10 μM CL treatment (Figure 4E and F), suggesting that the role of PKA in the CL-induced signaling cascade is intact in Epac1^-/-^ WAT. In addition to residue Ser660, HSL contains other phosphorylation sites regulated by ERK1/2 and AMPK. ERK1/2 is shown to be required to activate the maximal lipolytic activity, in addition to PKA-mediated lipolysis [17]. On the contrary, AMPK-mediated phosphorylation of HSL is reported to exert an anti-lipolytic effect [18]. In both Epac1^+/+^ and Epac1^-/-^ WAT explants treated with CL, the ratio of phospho-ERK1/2 (pERK1/2) to total ERK1/2 was increased (Figure 4G and H), demonstrating that ERK1/2 activation induced by CL is independent of Epac1. In addition, phospho-AMPK was not altered in WAT explants treated by CL in both genotypes (Figure 4I and J).

### Cold-induced thermogenic and lipolytic responses are impaired in Epac1^-/-^ mice

**Figure 5:**
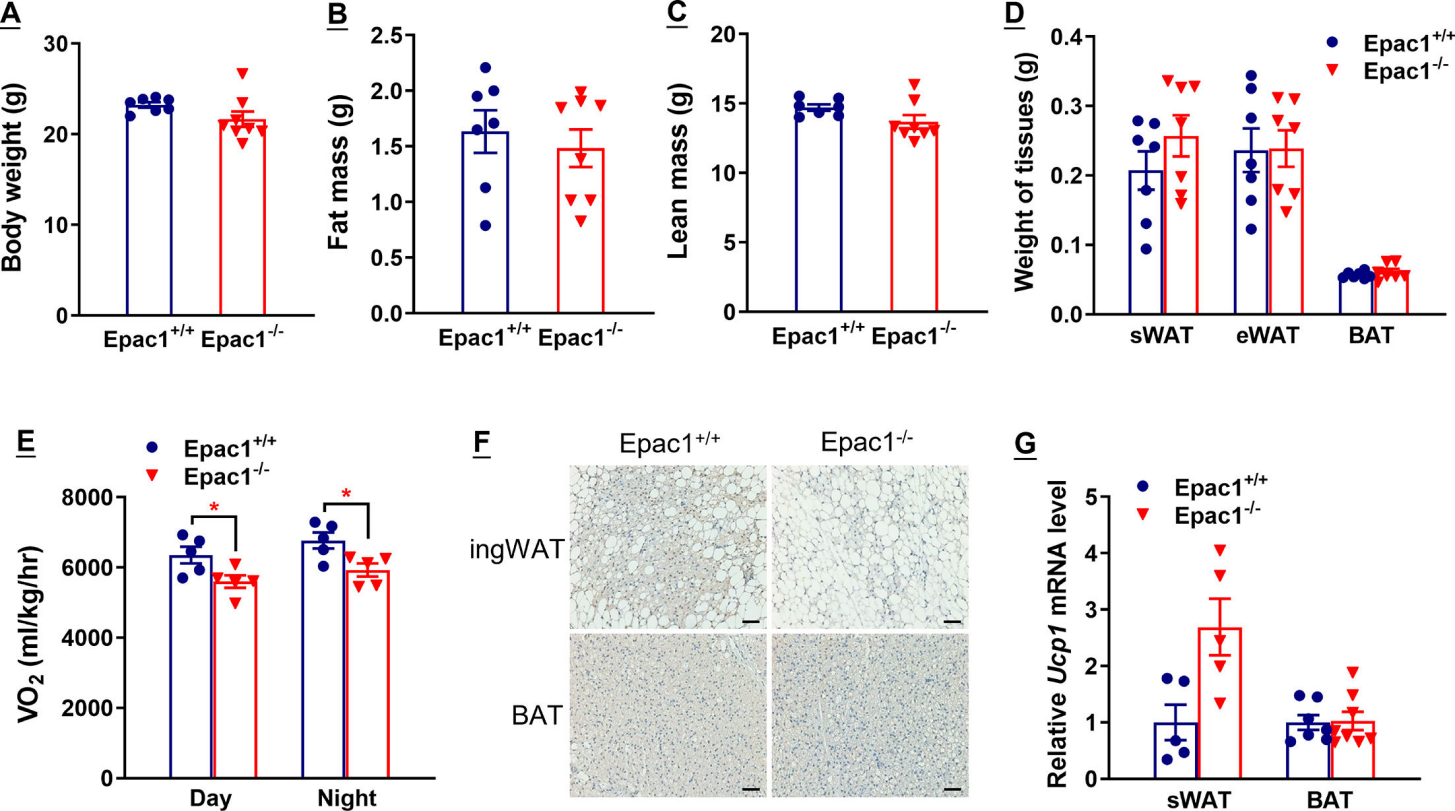
**Cold-induced beiging of ingWAT is impaired in Epac1^-/-^ mice**. Eight-week-old Epac1^+/+^ and Epac1^-/-^ mice were housed in a thermoneutral environment (30°C) for three weeks and then subjected to exposure at 6°C for 48 hours. (**A**) Body weight. (**B-C**) Fat mass (**B**) and lean mass (**C**) determined by a nuclear magnetic resonance (NMR) body composition analyzer. (**D**) Wet weight of ingWAT, eWAT, and BAT. (**E**) Oxygen consumption determined by metabolic cages. (**F**) Immunohistochemical staining of UCP1 in ingWAT and BAT. Scale bar, 50 μm. (**G**) mRNA level of UCP1 in ingWAT. Data are presented as mean ± SEM. *P<0.05. *n* = 5–8 for each group. BAT, brown adipose tissue; WAT, white adipose tissue.

With similar functions as the pharmacological β3-AR agonist CL, cold exposure is a key environmental stimulus of β3-AR, which stimulates the sympathetic nervous system to release norepinephrine and subsequently activates β3-AR signaling, thereby controlling the thermogenic and lipolytic responses [28]. To investigate whether Epac1 mediates cold-induced adaptive thermogenesis, Epac1^+/+^ and Epac1^-/-^ mice after three-week thermoneutralization were subjected to 48 hour cold exposure. There was no obvious difference in body weight, body composition, and weight of adipose tissues between Epac1^+/+^ and Epac1^-/-^ mice (Figure 5A–D). While consistent with what we observed in CL-treated mice, energy expenditure and cold-induced beiging were significantly abolished in Epac1^-/-^ mice, as evidenced by decreased whole-body oxygen consumption (Figure 5E) and less UCP1-positive multilocular beige adipocytes in ingWAT (Figure 5F). Additionally, a slight increase in UCP1 mRNA level was observed in ingWAT of Epac1^-/-^ mice, which was considered a compensatory effect in response to the decreased UCP1 protein level (Figure 5G).

**Figure 6:**
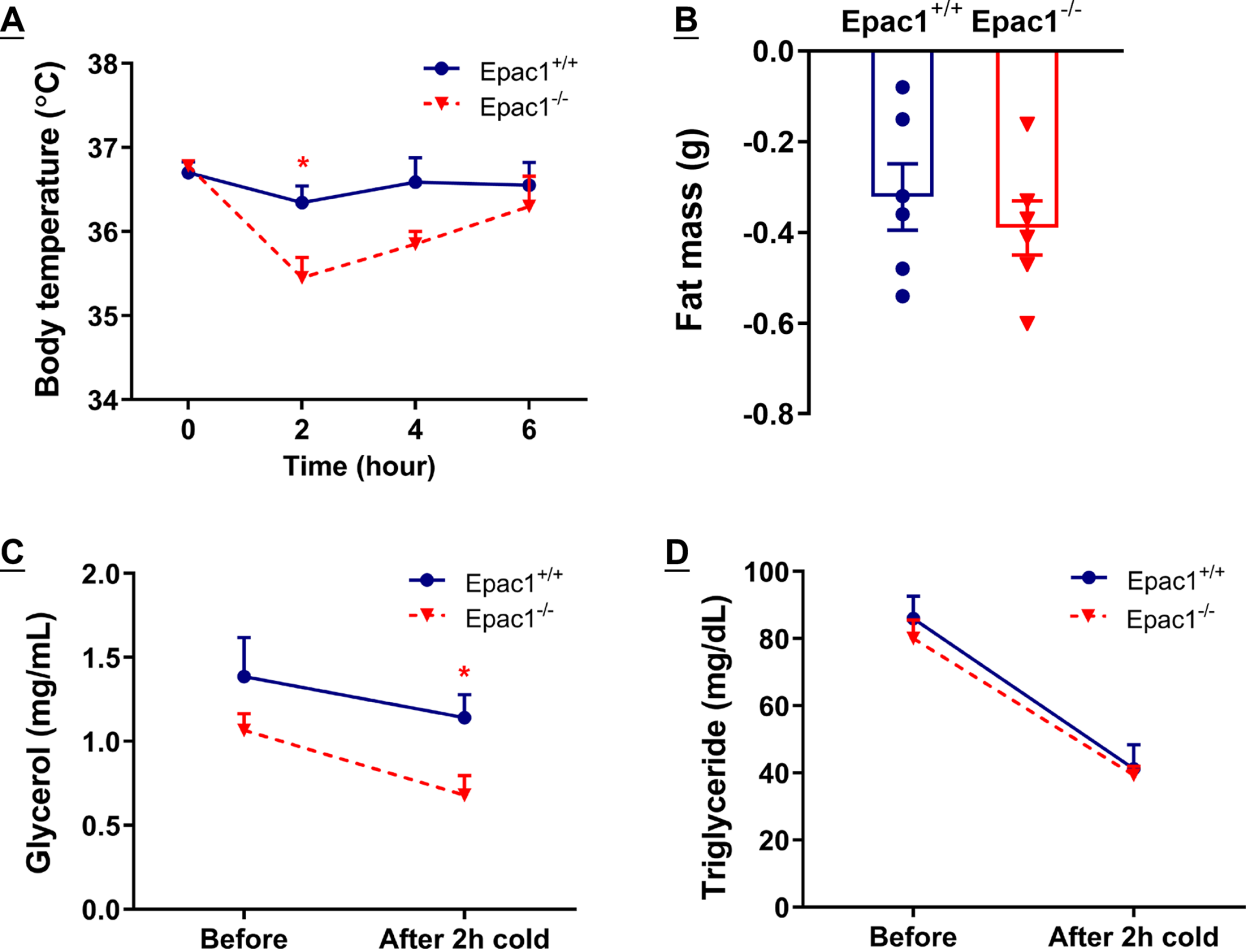
**Epac1^-/-^ mice display defective thermogenic and lipolytic responses upon acute cold stimulation**. Eight-week-old Epac1^+/+^ and Epac1^-/-^ mice were housed at thermoneutral environment (30°C) for three weeks and then subjected to exposure at 6°C for 6 hours. (**A**) Core body temperature during 6 hour cold challenge. (**B**) Change of fat mass. (**C**) Circulating glycerol level. (**D**) Circulating triglyceride level. Data are presented as mean ± SEM. *P<0.05. *n* = 3–6 for each group.

In addition to the long-term cold-induced beiging, we explored the short-term cold-induced thermogenic and lipolytic responses by subjecting Epac1^+/+^ and Epac1^-/-^ mice to 6 hour acute cold exposure. We found that the core body temperature of Epac1^-/-^ mice was significantly lower than Epac1^+/+^ mice after cold exposure for 2 hours (Figure 6A). Despite the similar reductions in whole-body fat content and circulating triglycerides, circulating glycerol levels of Epac1^-/-^ mice were markedly lower than the Epac1^+/+^ controls after 2 hour cold challenge (Figure 6B–D). These data collectively indicate that Epac1 mediates thermogenic and lipolytic responses upon both acute and chronic cold stimulation.

### Epac1^-/-^ WAT shows lower expression of transcriptional activators for UCP1 and decreased total and phosphorylated p38γ

**Figure 7:**
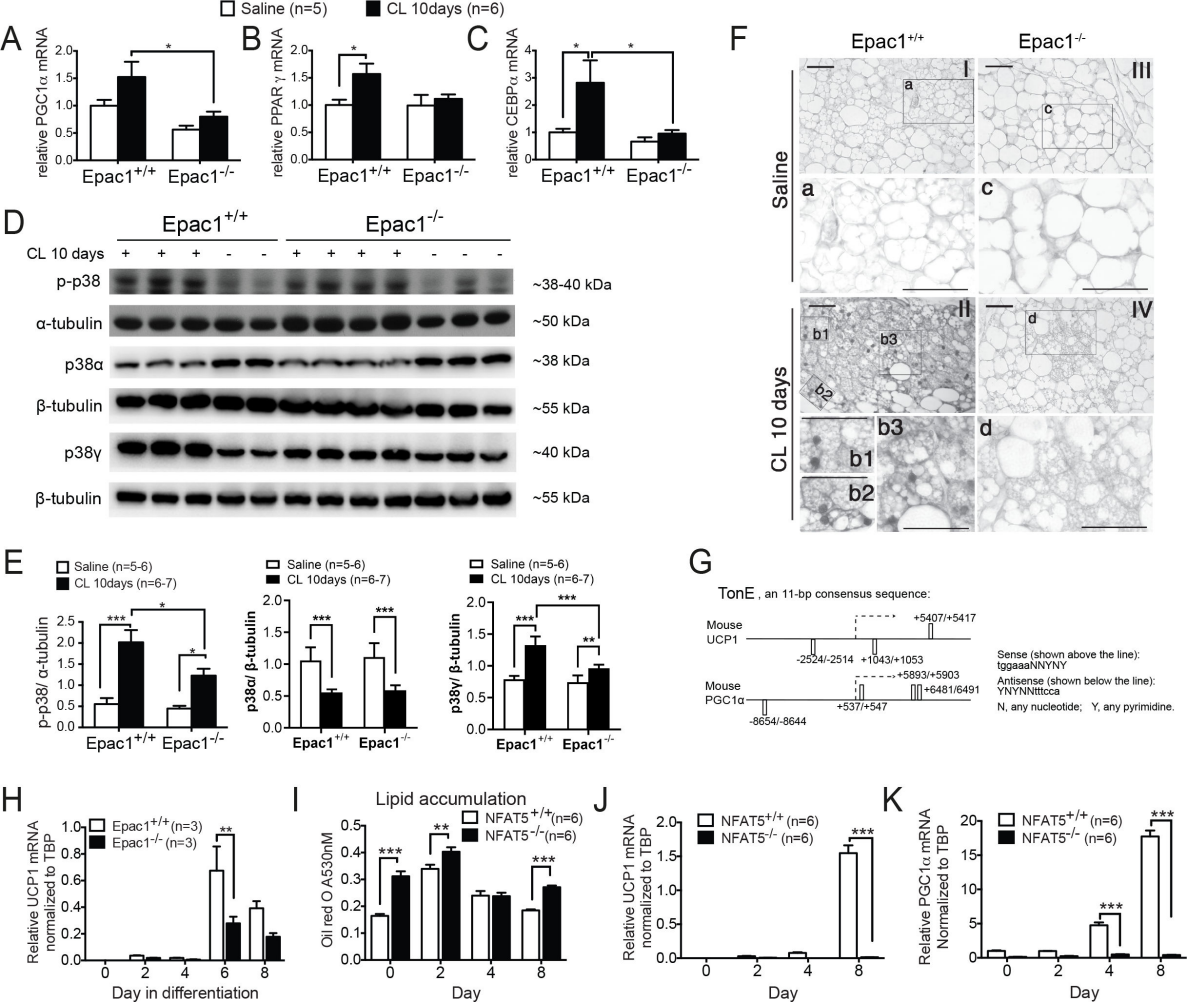
**Epac1 regulates thermogenic genes transcription**. (**A-C**) qPCR analysis (*n* = 5–6) of PGC1α (**A**), PPARγ (**B**), and CEBPα (**C**). (**D-E**) Western blot analysis of phospho-p38, total p38α, and total p38γ in WAT from mice treated with saline or CL for 10 days (*n* = 5–7). (**F**) Immunostaining of NFAT5 in WAT after saline or CL treatment for 10 days (*n* = 4–5). (**G**) Putative TonE sites in mouse UCP1 and PGC1α genes. (**H**) UCP1 mRNA expression in Epac1^+/+^ and Epac1^-/-^ adipocytes derived from MEF (*n* = 3). (**I-K**) Adipocytes differentiation of NFAT5^+/+^ and NFAT5^-/-^ MEF (*n* = 6). (**I**) Quantitative analysis of Oil red O staining on differentiation day 0, 2, 4 and 8 (*n* = 6). Induction of UCP1 (**J**) and PGC1α (**K**) mRNA during the differentiation (*n* = 6). Data are presented as mean ± SEM. *P<0.05, **P<0.01, ***P<0.001. MEF, mouse embryonic fibroblast; NFAT5, nuclear factor activated in T cells 5; WAT, white adipose tissue.

Many BAT-enriched proteins are demonstrated to regulate UCP1 transcription and brown adipocyte differentiation. For example, peroxisome proliferator-activated receptor gamma (PPARγ) and CCAAT/enhancer binding protein alpha (C/EBPα) play fundamental roles in adipocytes differentiation [29,30]. As a nodal regulator of mitochondrial biogenesis, PPARγ coactivator 1-alpha (PGC1α) up-regulates UCP1 [31] and initiates thermogenesis. Increased mRNA expression of these BAT-enriched genes was observed in the WAT of CL-treated Epac1^+/+^ mice, while it is compromised in Epac1^-/-^ mice (Figure 7A–C), supporting the notion that Epac1 plays a critical role in CL-induced up-regulation of UCP1 and thermogenesis. It has been reported that phosphorylation of p38 MAPK is required for regulating transcription of UCP1 upon β3-AR activation in primary brown adipocytes [10]. More importantly, different subtypes of p38 MAPK, namely p38α and p38γ/δ, are shown to have opposite roles in adipose tissues, in which p38γ/δ promotes thermogenesis, while p38α inhibits it [32]. Therefore, we tested total p38α and p38γ levels together with p38 MAPK phosphorylation in WAT and investigated their changes in the presence and absence of Epac1. The basal levels of phospho-p38 MAPK, total p38α, and total p38γ in peri-uterine WAT were similar between Epac1^+/+^ and Epac1^-/-^ (Figure 7D and E). After 10 days of CL treatment *in vivo*, both Epac1^+/+^ and Epac1^-/-^ peri-uterine WAT showed increased p-p38 MAPK and total p38γ levels but decreased total p38α levels (Figure 7D and E). Nevertheless, Epac1^-/-^ WAT showed a significantly smaller increase in p-p38 MAPK and total p38γ but similar reduction of total p38α when compared with the Epac1^+/+^ group (Figure 7D and E). Notably, the upper band in the Western blot analysis for phospho-p38 corresponds to the molecular weight of p38γ (Figure 7D), thus indicating that Epac1 contributes at least in part to the regulation of total and phosphorylated p38γ but not p38α after β3-AR activation.

### CL-induced NFAT5 expression in the nuclei of beige cells is lower in the absence of Epac1

Next, we asked what the downstream signal is following p38γ MAPK activation induced by CL treatment. NFAT5 is a transcription factor activated by p38 MAPK when a cell is exposed to hyperosmotic stress [33]. The activated NFAT5 translocates to the nucleus and binds to tonicity-responsive enhancers (TonE). Consequently, this leads to increased gene expressions involved in the production and uptake of organic osmolytes. A scaffold protein that binds to and regulates the GTPase Rac and upstream kinases in the p38 MAPK phospho-relay module has been discovered previously [34]. Given the important role of Epac1 in regulating Rac GTPase and downstream signaling [35], we hypothesized that the NFAT5 pathway might be defective in the Epac1^-/-^ mice due to reduced CL-induced phosphorylation of p38γ MAPK. It prompted us to examine NFAT5 in the adipose tissue in the Epac1^-/-^ mice. Immunostaining of NFAT5 on the membrane or cytoplasm of all white adipocytes was shown in both genotypes, suggesting NFAT5 was located in the cytoplasm of white adipocytes (Figure 7F I and III). Moreover, prominent immunostaining of NFAT5 was observed in both the cytoplasm and the circular nuclei in the beige fat area in Epac1^+/+^ mice treated with CL (Figure 7F II). These cells shared a similar morphology with the UCP1-positive beige cells (Figure 7F II, see bottom figures for enlargement of framed areas b1-b3 in top figure). In contrast, in the WAT of CL-treated Epac1^-/-^ mice, NFAT5 staining in the cytoplasm was less intense, and there was very little staining in the nuclei of the multilocular cells (Figure 7F IV and d). These results suggest that Epac1 plays a role in regulating the expression and/or nuclear translocation of NFAT5 in the beige adipocytes. In addition, several TonE consensus sequences (5′-TGGAAANNYNY-3′) in both orientations were found in mouse UCP1 and PGC1α genes (Figure 7G).

### Both Epac1 and NFAT5 are critical in adipocyte differentiation and UCP1 induction *in vitro*

It has been shown that UCP1 expression can be induced in adipocytes derived from MEFs [36]. Insulin sensitizer PPARγ agonist was reported to increase UCP1 and mitochondrial biogenesis and remodeling during adipogenesis [37,38]. To further test whether Epac1 plays a role in UCP1 induction and adipocyte differentiation, we differentiated Epac1^+/+^ and Epac1^-/-^ MEFs into adipocytes by supplementing classic adipogenic agents: dexamethasone, insulin, IBMX, and a PPARγ agonist pioglitazone. In line with our *in vivo* findings on UCP1 expression as well as the *in vitro* findings during SVF differentiation, levels of UCP1 mRNA expression were lower in adipocytes derived from Epac1^-/-^ MEF compared to those of the Epac1^+/+^ on day 6 (Figure 7H). This suggested that Epac1 mediates UCP1 transcription upon stimulation by PPARγ agonist and adipogenic agents. To further test the role of NFAT5 in UCP1 induction and adipocyte differentiation, the same experiment was also performed using immortalized NFAT5^+/+^ and NFAT5^-/-^ MEFs [39]. In addition, NFAT5^-/-^ MEF-derived adipocytes showed more lipid accumulation on day 0, 2, and 8, compared with that of the NFAT5^+/+^ (Figure 7I). Interestingly, NFAT5^-/-^ adipocytes expressed significantly lower UCP1 and PGC1α transcript levels (Figure 7J and K), suggesting the role of NFAT5 in the transcription of thermogenic genes. Taken together, Epac1 regulates the expression and nuclear translocation of NFAT5, which further mediates the transcription of UCP1 and PGC1α in beige adipocytes.

### Discussion

**Figure 8:**
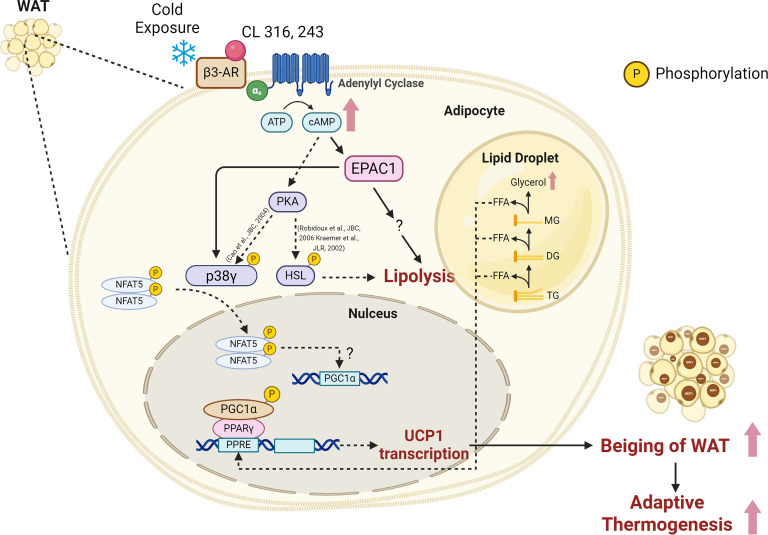
Epac1 mediates CL316,243- and cold-induced thermogenesis and lipolysis in WAT by modulating the p38γ-NFAT5 axis in a PKA-independent manner.

We report here that Epac1^-/-^ mice show reduced thermogenesis and lipolysis upon CL- and cold-induced β3-AR activation. Administration of CL to Epac1^-/-^ mice for 10 days or cold challenge for 48 hours was unable to induce the development of beige cells with UCP1 expression in WAT. Deletion of Epac1 also led to impaired β3-adrenergic induction of lipolysis upon CL treatment or cold exposure, resulting in a lower substrate supply for thermogenesis. Additionally, *in vitro* beige adipogenesis was significantly impaired in Epac1-deficient mice, and cAMP analog-induced beige differentiation was also abolished in Epac1^-/-^ mice. Mechanistically, the decreased mRNA levels of CL-induced thermogenic genes UCP1, PGC1α, and PPARγ in WAT of Epac1^-/-^ mice could be explained by the concomitant reduction in total and phosphorylated p38γ, together with the downstream nuclear translocation of NFAT5. However, there is no obvious difference in PKA-activated phosphorylation of HSL in ingWAT between Epac1^+/+^ and Epac1^-/-^ mice, and treatment with a PKA activator has no impact on *in vitro* beige adipocyte biogenesis in Epac1^-/-^ mice, thus highlighting the PKA-independent mechanism of Epac1 actions on lipolysis and beige adipogenesis. Taken together, Epac1 mediates β3-AR induced beiging and lipolysis of WAT via the p38γ-NFAT5 axis in a PKA-independent manner (Figure 8).

The defective beiging of WAT can be attributed primarily to the attenuated CL- and cold-induced thermogenesis and energy expenditure in Epac1^-/-^ mice. This is because no prominent changes were observed in the morphology, weight, and UCP1 transcript levels in BAT upon β3-AR activation compared to those of the wild-type mice. As the key molecule in the autonomic regulation of energy expenditure and adiposity, UCP1 is indispensable in the anti-obesity effect of β3-adrenergic stimulation [40]. A previous study excluded the role of Epac1 in β3-adrenergic activation by showing little induction of GTP-Rap1 in the Rap1 pull-down assay after β3-AR activation for 5 minutes *in vitro* [41]. While in this study, we elucidated that Epac1 is essential for beiging of WAT by comparing the Epac1^-/-^ mice and their wild-type littermates under prolonged β3-adrenergic activation conditions (treated with CL or cold exposure). Lower levels of UCP1 in Epac1^-/-^ MEF-derived adipocytes stimulated by PPARγ agonist also suggest that Epac1 is involved in the regulation of UCP1 gene expression. This finding came to the same conclusion as the study by Reverte-Salisa et al., who treated brown preadipocytes with 007, a small molecule activator of Epac1, and induced differentiation and up-regulated expression of UCP1 were observed [42]. Except for the similar observations in terms of the beiging of WAT as Reverte-Salisa et al. [42], our current study also demonstrates the involvement of the p38γ-NFAT5 axis in the regulation of Epac1-mediated lipolysis in a PKA-independent manner, thus leading to a more comprehensive understanding of non-PKA-regulated lipolytic pathways and also highlighting the multifaceted roles of Epac1 in both thermogenesis and lipolysis. In accordance with previous *in vitro* studies [43], CL-induced phosphorylation of p38 MAPK *in vivo* was decreased in the absence of Epac1.

On the other hand, FFA released from accelerated lipolysis serves as a fuel and intracellular signal for the uncoupling of electron transport from oxidative phosphorylation, consequently activating thermogenesis [44]. Additionally, lipolysis plays a critical role in the β3-adrenergic remodeling of WAT [45]. Here, we show that Epac1 deficiency impairs β3-adrenergic lipolysis in WAT *in vivo* and *ex vivo*, suggesting that Epac1 is one of the key regulators in cAMP-mediated lipolysis. Given the dramatic rise of FFA levels during cold exposure, the diet-induced BAT thermogenesis, and that fatty acids are considered as endogenous activators of PPAR [29], the reduced induction of UCP1, thermogenesis, and energy expenditure in Epac1^-/-^ mice could be the outcome of impaired β3-AR activated lipolysis and the subsequent reduction in PPAR activation. It has been suggested that β3-AR activation of lipolysis is mediated through both cAMP-dependent PKA and ERK [17]. In the current study, however, the reduced lipolysis in Epac1^-/-^ WAT is unlikely due to defective PKA signaling, given that the PKA-mediated phosphorylation site on the HSL was not altered in Epac1^-/-^ WAT. Further studies are needed to understand the mechanism of impaired β3-adrenergic lipolysis in the absence of Epac1.

P38 MAPK family contains four different proteins—p38α, p38β, p38γ, and p38δ—which are not isoforms but distinct proteins with different substrates and distinct roles in different tissues, including muscle, adipose tissue, and heart [32,46,47]. Here, we show that Epac1 mainly regulates p38γ but not p38α, which is also in line with the previous report showing opposite roles of p38α and p38γ/δ in regulating thermogenesis in adipose tissues [32]. Our previous study established that NFAT5 activation under hyperosmotic stress requires p38 MAPK activation [33], and a more recent study further demonstrated that a GTPase Rac complex aids the activation of p38 MAPK [34]. It was proposed that the NFAT family of transcriptional regulatory proteins plays a potential role in adipogenesis [48]. Recently, the mRNA of NFAT5 is detected in epididymal, perirenal, and BATs [49]. NFAT5 was also identified as an interacting partner to fat-specific protein 27 (FSP27, also called Cidec), a lipid droplet (LD)-associated protein that induces the accumulation of LDs [49]. Although a previous report shows an inhibitory effect of NFAT5 on beiging of WAT to promote obesity [50], our present findings demonstrate that NFAT5 acts downstream of Epac1 to positively regulate UCP1 transcription during beiging *in vivo* and beige adipogenesis *in vitro*, suggesting that NFAT5 might have opposite effects upon differential upstream regulations. Therefore, we proposed a novel mechanism in which Epac1 plays a crucial role in regulating β3-AR stimulated UCP1 transcription, possibly by mediating p38γ and NFAT5 activation.

Previously, we demonstrated that Epac1^-/-^ mice show normal food intake and similar levels of leptin during regular diet and high-fat diet feeding, indicating that leptin signaling and feeding behavior in the Epac1^-/-^ mice was not affected [20]. In this study, we show the induction of UCP1 expression in WAT after 10 days of CL treatment is dramatically reduced in the Epac1-deficient mice. Accordingly, the impairment of β3-adrenergic induction of UCP1 expression in WAT may explain the higher TG level and the more severe high-fat diet-induced obesity in Epac1^-/-^ mice [20]. The larger size of adipocytes in WAT in high-fat diet-fed Epac1^-/-^ mice (data not shown) also indicates the role of Epac1 in lipid metabolism.

Taken together, the current study provides novel insights into the mechanism of β3-AR activation induced thermogenesis and lipolysis, as well as new pathways for pharmaceutical intervention of obesity. Our findings illustrate that CL- and cold-induced β3-AR activation increases lipolysis and beiging of WAT in an Epac1-dependent manner, in which Epac1 plays essential roles in regulating UCP1 transcription and PKA-independent lipolysis, possibly through the p38γ-NFAT5 axis. The regulation of lipolysis and beiging of WAT is a complex process, and further studies will be needed to provide more intensive mechanistic insights into the regulation of β3-AR-induced lipolysis and thermogenesis via the Epac1-p38γ-NFAT5 axis.

**Clinical Perspectives**

- Boosting thermogenesis (beiging) and lipolysis in white adipose tissue (WAT) is considered as a promising therapeutic strategy to counteract obesity and its related metabolic disorders. The cAMP-mediated protein kinase A (PKA) functions in metabolism are well-documented, while the role of PKA-independent metabolic pathways is still poorly understood.
- Epac1, a downstream target of cAMP, mediates β3-AR activation to modulate thermogenesis and lipolysis in white adipose tissue (WAT) in a PKA-independent manner. Both CL316,243- and cold-induced beiging and lipolytic response of WAT are compromised in Epac1-deficient mice, which is attributed to the impaired UCP1 transcription and PKA-independent lipolysis driven by reduced p38γ and decreased induction of NFAT5.
- Our study provides novel insights into a PKA-independent mechanism of β3-AR activation induced thermogenesis and lipolysis, as well as new pathways for pharmaceutical intervention of obesity.

## Data Availability

The data that support the findings of this study are available from the corresponding author upon reasonable request.

## Abbreviations

AMPK: AMP-activated protein kinase
BAT: brown adipose tissue
C/EBPα: CCAAT/enhancer binding protein alpha
CL: CL316,243
ERK: extracellular signal-regulated kinase
Epac1: exchange protein directly activated by cAMP 1
FFA: free fatty acid
HSL: hormone sensitive lipase
MAPK: mitogen-activated protein kinase
MEF: mouse embryonic fibroblast
NFAT5: nuclear factor activated in T cells 5
PGC1α: PPARγ coactivator 1-alpha
PKA: protein kinase A
PPARγ: peroxisome proliferator-activated receptor gamma
SVFs: stromal vascular fractions
TBP: TATA-box binding protein
TonE: tonicity-responsive enhancers
UCP1: uncoupling protein 1
WAT: white adipose tissue
β3-AR: β3-adrenergic receptor
